# KCa3.1 mediates radioresistance of silver nanoparticles in human glioblastoma cells

**DOI:** 10.1007/s00424-026-03179-8

**Published:** 2026-05-20

**Authors:** Francesco Ragonese, Andrea Biagini, Luana Sallicandro, Alessandra Mirarchi, Gianmarco Reali, Antonella De Luca, Cataldo Arcuri, Sara Flamini, Oxana Bereshchenko, Paolo Cogliati, Roberto Spogli, Paola Sabbatini, Stefano Bruscoli, Paolo Sportoletti, Bernard Fioretti

**Affiliations:** 1 Department of Chemistry, Biology and Biotechnology, University of Perugia, Perugia, 06123 Italy; 2 Department of Medicine and Surgery, University of Perugia, Perugia, 06132 Italy; 3 Prolabin & Tefarm, Spin-Off University of Perugia, Perugia, 06134 Italy; 4 Department of Medicine and Surgery, Institute of Hematology, Centro di Ricerca Emato-Oncologica (CREO), University of Perugia, Perugia, 06129 Italy

**Keywords:** Silver nanoparticles, Ion Channels, Glioblastoma, Nanomedicine, Radioresistance, Intracellular calcium, KCa3.1

## Abstract

Silver nanoparticles (AgNPs) have emerged as a promising therapeutic tool for managing glioblastoma (GB), particularly in radiosensitisation. However, their mechanisms of action are not yet fully understood. Since ion channel activity is implicated in various aspects of radioresistance, we investigated the electrophysiological effects of AgNPs on GB cells. In the U251 human GB cell line, the acute application of AgNPs induced the activation of a cationic nonselective current in a dose-dependent manner, which was not affected by ACA (TRPM2 blocker). In addition, AgNPs increased intracellular calcium concentration, caused depolarisation of the mitochondrial inner membrane, and promoted membrane blebbing. The investigation revealed that calcium influx is a critical step in the AgNPs-induced effects. Thiol reagents such as cysteine are known silver chelating agents and their application prevented the activation of cationic currents, intracellular calcium increases, and membrane blebbing formation induced by AgNPs. This finding suggested Ag^+^ are the active species responsible for these effects, acting through IAg current previously characterized in our laboratory. Intracellular calcium influx induced by AgNPs activated intermediate-conductance calcium-activated potassium currents. This activation was accordingly blocked by TRAM-34, a selective inhibitor of KCa3.1 channels. Finally, AgNPs were found to reduce U251-GB cell viability in a dose-dependent manner and to increase G2/M phase accumulation following sublethal irradiation. In the presence of TRAM-34 the G2/M phase accumulation induced by the combined treatment with AgNPs and irradiation was reduced, suggesting the involvement of radioresistance-associated KCa3.1 currents. These results provide new insights into optimizing the use of AgNPs as radiosensitizing agents in GB therapy.

**Supplementary Information:**

The online version contains supplementary material available at 10.1007/s00424-026-03179-8.

## Introduction

Glioblastoma (GB) is one of the most malignant types of central nervous system tumour in humans and is characterized by a very low life expectancy with a mean survival of about 15 months from diagnosis [1]. The poor prognosis is due to its invasiveness, metastatic behaviour, intra- and intertumoral heterogeneity, immunosuppressive microenvironment and resistance to radio and chemo-treatments [2, 3]. In recent years, several therapeutic approaches have been attempted, such as chemotherapy, tomoradiotherapy, electro-modulated or nanoparticle-based hyperthermia, immunotherapy, virotherapy and gene therapy [4, 5]. Despite the extensive research conducted on this neoplasm, only a modest increase in life expectancy has been observed. At present, the standard protocol for the treatment of GB is neurosurgical treatment following a combination of radiotherapy and temozolomide (TMZ), a radiosensitizing chemotherapeutic agent [6, 7].

Silver nanoparticles (AgNPs) have a wide range of biomedical applications including cancer therapy, due to their ability to target tumor tissue cells and to their intrinsic cyto- and genotoxicity based on reactive oxygen species (ROS) production, caspases activation, mitochondrial dysregulation, and induction of apoptosis and autophagy [8–10]. AgNPs are known to be radiosensitizing agents against GB both in vitro and in vivo [11–13]. Several studies have demonstrated an increase in radiation-induced apoptosis and autophagy in various glioma and GB cell lines, including murine C6 and human U251 [14, 15]. Multiple studies have demonstrated that AgNPs induce genotoxic effects in glioblastoma cells, including U251. AshaRani et al. reported dose-dependent ROS generation, mitochondrial dysfunction, and significant DNA damage in U251 cells, detected by comet and micronucleus assays [8]. Subsequent analyses showed activation of DNA damage response pathways, including γH2AX foci formation, modulation of DNA repair genes, G2/M arrest, and p53 activation, supporting a ROS-driven mechanism of genomic instability [16]. Furthermore, AgNPs have been described as radiosensitizers in glioma models. Liu et al. demonstrated that AgNPs enhance radiation-induced cytotoxicity through increased oxidative stress and amplified DNA damage [11]. Collectively, these findings indicate that AgNPs-induced DNA damage in glioblastoma is largely mediated by oxidative stress and DDR activation.

Other works shown that AgNPs can also enhance the drug sensitivity to TMZ, resulting in a stronger antiproliferative effect [17, 18]. Some of the anticancer properties of AgNPs have been related to the release of silver ions (Ag^+^) from the metal core of the nanoparticles, caused by surface oxidation [19]. Free Ag^+^ released from AgNPs is involved in the destruction of cellular membranes [20], in bleb formation [21], DNA damage [22] and neurotoxicity [23].

The intermediate-conductance calcium-activated potassium channel (KCa3.1, gene *KCNN4*) is expressed in GB, and its expression correlates with clinicopathological features and patient prognosis [24]. Our research group was the first to report the expression of these channels in human glioblastoma multiforme [25]. These channels have been implicated in migration/invasion processes [26, 27] and in the development of radioresistance in GB cells both in vitro and in vivo [28–31]. Intracellular calcium increase represents the main activation signal of this channel also in GB [26, 32]; however, regulation which depends on phosphorylation events of KCa3.1, induced by radiosensitizer agent LY294002, has also been described in GB [33].

The involvement of KCa3.1 in AgNPs-mediated radiosensitization/radioresistance has never been investigated, and this information could be crucial for the development of new radiotherapeutic protocols exploiting AgNPs. We report that AgNPs activate a nonselective cationic current (IAg) permeable to calcium through the release of silver ions. The increase in intracellular calcium, in turn, activates the calcium-activated potassium current. Notably, the G2/M accumulation observed after co-treatment with IR and AgNPs was reduced by the radiosensitizer TRAM-34, a selective blocker of intermediate-conductance calcium-activated potassium channels [31]. These results demonstrate that the activation of IAg and KCa3.1 currents represent an early event that should be considered in order to optimize the radiosensitizing effects of AgNPs in GB.

## Materials and methods

### Synthesis and characterization of AgNPs

AgNPs were synthesized in H_2_O by a slight modification of the Agnihotri method [34]. All chemicals were of analytical grade. An aqueous solution of trisodium citrate (2.5 mM, 10 mL) and AgNO_3_ (2.5 mM, 10 mL) was prepared in a three-neck round bottom flask under vigorous stirring. After 5 min, an aqueous solution of NaBH_4_ (0.104 M, 0.72 mL) was added dropwise to the solution and the mixture was stirred for 30 min at room temperature. The colloid suspension was centrifuged at 30.000 g in presence of 0.025% Tween20 (Invitrogen) as stabilizer and then the pellets were resuspended at the desired concentration in 1 mM citrate solution. For size characterization, Transmission Electron Microscopy (TEM) analysis was performed using a Philips 208 microscope, operating at an accelerating voltage of 100 kV. AgNPs pure stock was diluted 1:1000 with an aqueous solution citrate (1 mM). A drop of colloidal suspension was poured on a support grid and evaporated in a vacuum dryer.

### Zeta potential measurement

The electrokinetic properties of AgNPs were evaluated by electrophoretic light scattering (ELS) using a Litesizer DLS 500 (Anton Paar, Austria). Measurements were performed at 25.0 °C using disposable folded capillary cells. Samples were diluted 1:8 in ultrapure water prior to analysis to avoid multiple scattering effects and minimize conductivity artifacts. The ζ-potential was calculated from electrophoretic mobility values using the Smoluchowski approximation (Henry function f(κa) = 1.5), appropriate for aqueous media of moderate ionic strength. For each sample, at least three independent measurements were performed, and results are reported as mean ± standard deviation. Conductivity was recorded during acquisition to ensure measurement consistency.

### Cell culture

U251 cells were purchased from Cell Lines Service GmbH (Eppelheim, Heidelberg, Germany) and were grown in Dulbecco’s Modified Eagle Medium (DMEM) supplemented with 10% heat-inactivated Fetal Bovine Serum (FBS), 100 IU/ml penicillin/streptomycin, and 200 mM of L-glutamine. The flasks were incubated at 37 °C in a 5% CO_2_-humidified atmosphere. The medium was changed twice a week, and the cells were sub-cultured when confluent. For Ca^2+^ imaging, electrophysiology and blebbing imaging experiments, 3 × 10^4^ cells were plated in a 35 mm Petri dish and used on the third day of culture. For all other experiments, 1 × 10^4^ cells/cm^2^ were plated and used/treated on the second day of culture. All cell culture supports, plates and flasks were purchased from Falcon (Corning). Pictures of cells were taken using an Axio Examiner (Zeiss) with charge-coupled device (CCD) Axiocam 502 mono digital camera (Zeiss).

### Electrophysiological recordings

Whole-cell perforated patch-clamp configuration was used for electrophysiological recordings from U251 cells. Currents and voltages were amplified with a HEKA EPC-10 amplifier and analysed with PatchMaster and Origin 4.1 software. For online data collection, currents were filtered at 3 kHz and sampled at 100 µsec/point. Membrane capacitance measurements were made by using the transient compensation protocol of PatchMaster. The external solution contained (in mM): NaCl (106.5), KCl (5), CaCl_2_ (2), MgCl_2_ (2), MOPS (5), glucose (20), Na gluconate (30), pH 7.25. Octanol (1 mM), triarylmethane-34 (TRAM-34, 3 µM) and tetraethylammonium chloride (TEA, 3 mM) were added to the external bathing solution to block gap-junctions, Intermediate and Big conductance Ca^2+^-activated potassium channels, respectively [32, 35]. The pipette solution contained (in mM) K_2_SO_4_ (57.5), KCl (54), MgCl_2_ (5), MOPS (10), pH 7.20. Amphotericin B (200 µM) was added to the pipette solution to achieve electrical access to the cytoplasm ranging between 10 and 20 MΩ within 10 min after seal formation. Cells were continuously perfused using a gravity-driven perfusion system, focally oriented onto the field of interest. Solutions with AgNPs were prepared immediately before application by diluting the AgNPs stock with external solutions. 4,4′-Diisothiocyanatostilbene-2,2′-disulfonic acid (DIDS), N-(p-amylcinnamoyl) anthranilic acid (ACA) were obtained from Sigma-Aldrich (Merck, Germany).

### FURA-2 calcium imaging

Before experiments, cells were incubated with 3 µM of FURA-2-AM (Merck) for 45 min and extensively washed with Physiological Salt Solution (PSS) of following composition (in mM): NaCl (140), KCl (5), CaCl_2_ (2), MgCl (2), MOPS (5), glucose (10), at pH 7.4 as described in Ragonese et al., 2019 [35]. Cells were continuously perfused using a gravity-driven perfusion system, focally oriented onto the field of interest. The intracellular free calcium concentration was estimated by analyzing the change in the fluorescence emission ratio at 510 nm, obtained using excitation wavelengths of 340 and 380 nm (optical filters and dichroic beam splitter from Lambda DG4, Shutter Instruments). Ratiometric data were acquired every 3 s and fluorescence determinations were performed using fluorescence microscopy system Zeiss (Axio Examiner V16 and Axiocam 502 mono). The acquisition and analysis were driven by ZEN 2 software (Zeiss). The intracellular calcium was estimated as percentage of change of the 340/380 ratio.

### Morphological and TEM analysis

Bleb formation was examined after washing the cultured U251 cells twice and perfusing them in Ringer’s solution. Time-lapse pictures of cells were taken every 30 s at 20x magnification using an Axio Examiner V16 (Zeiss) with CCD Axiocam 502 mono digital camera (Zeiss) for at least 30 min. The measurement of blebs in terms of number and area was conducted using Zen 2 image processing software (Zeiss) as described in Ragonese et al., 2019 [35]. For TEM analysis, U251 cells were treated with 1.7 nM AgNPs for 24 h, then harvested, pelleted, and embedded in resin prior to ultrathin sectioning.

### Inner mitochondrial membrane potential analysis

Cultured cells were incubated with 30nM of tetramethylrhodamine methyl ester perchlorate (TMRM, Merck) for 30 min at 37 °C in DMEM and washed twice with Ringer’s solution after the staining as described in Ragonese et al., 2021 [36]. During experiments, cells were continuously perfused with Ringer using a gravity-driven perfusion system, focally oriented onto the field of interest. Time-lapse images were captured every 60 s using a fluorescence microscopy system Zeiss (Axiozoom V16 and Axiocam 502 mono) with a dichroic system and excitation/emission 545/605 nm wavelength filter set from Zeiss. Light source comes from an HXP 120 V illuminator (Zeiss). The variation of mitochondrial membrane potential (ΔΨ) was measured as the change of the emission fluorescence intensity during the time. Measurements were performed with ZEN 2 image analysis software.

### Cell viability assay

3-(4,5-Dimethylthiazol-2-yl)−2,5-diphenyltetrazolium bromide (MTT) cell assay was used to assess cell viability as described in Ragonese et al., 2021 [36]. For this purpose, 4 × 10^3^ cells were plated in 96-well and treated with AgNPs after 24 h after seeding. After 24 h and 48 h from treatment, cells were incubated with 0.4 mg/ml of MTT for 2.5 h. Then, the supernatant was removed and 200 µl of dimethyl sulphoxide (DMSO) were added into wells. Viability was analyzed at 550 nm wavelength using Multiskan MS (Labsystems). The experiments were repeated by culturing the cells in DMEM without FBS and penicillin/streptomycin. This was done immediately prior to the treatment with AgNPs. The results were expressed as normalized percentage, based on the ratio of the absorbance of treated cells to that of the controls.

### Cell cycle analysis

2.5 × 10^5^ cells were plated on 35 mm Petri dishes for 24 h and then treated with AgNPs and TRAM-34 and irradiated with 3.5 Gy of ionizing radiation (IR). After 24, cells were trypsinized, washed with ice-cold PBS, and resuspended in phosphate-buffered saline (PBS) plus 1 mg/mL of propidium iodide (PI). Cells were incubated with PI for 60 min at 4 °C and analyzed immediately after incubation. The DNA content was measured using a flow cytometer (FACS Calibur) with CellQuest software (Becton Dickinson). Recordings of at least 20,000 events was performed in each experiment/sample.

### Flow cytometric analysis of intracellular ROS production

U251 cells were seeded at a density of 5 × 10⁴ cells per well in 12-well plates (Thermo Fisher Scientific) and cultured in DMEM (Gibco) for 24 h at 37 °C in a humidified atmosphere containing 5% CO₂ Cells were washed twice with PSS and treated for a total of 40 min at 37 °C under the following conditions: (i) DMSO vehicle control (Thermo Fisher Scientific); (ii) 10 µM DCF-DA alone (Sigma-Aldrich, cat. no. 287810; added for the final 15 min of incubation); or (iii) 10 µM DCF-DA in combination with 1.7 nM AgNPs (30 min exposure). Following treatment, cells were detached using 1× Trypsin-EDTA (Aurogene) for 3 min at 37 °C, resuspended in Ringer’s solution, and centrifuged at 200 × g for 10 min at 4 °C. Cell suspensions were filtered through a 40 µm-cell strainer and maintained on ice in the dark until analysis. Intracellular ROS levels were quantified by flow cytometry using an Attune NxT cytometer (Thermo Fisher Scientific) with 488 nm excitation and fluorescence detection in the BL1 channel (530/30 nm). Data were analyzed using FlowJo software v10 (FlowJo LLC).

### Irradiation protocol

Cells were irradiated using an IBL 437 C Cesium 137 irradiator (CIS Bio International). Cells were exposed to a total IR dose of 3.5 Gy. Exposition time was determinate by calculating Cesium 137 decay time according to the manufacturer’s indications.

### Statistical analysis

All experiments were performed at least three times independently. Data are expressed as the mean ± standard deviation (SD), if not specified differently. Data were analysed by the unpaired two-tailed Student’s t-test or one-way ANOVA test. *p* < 0.05 (*), *p* < 0.01 (**), *p* < 0.001 (***) and *p* < 0.0001 (****) was used to assess the significance of the results. All statistical analyses were performed using Prism 7 Software and Origin Lab software.

## Results

### Synthesis and characterization of AgNPs

**Fig. 1:**
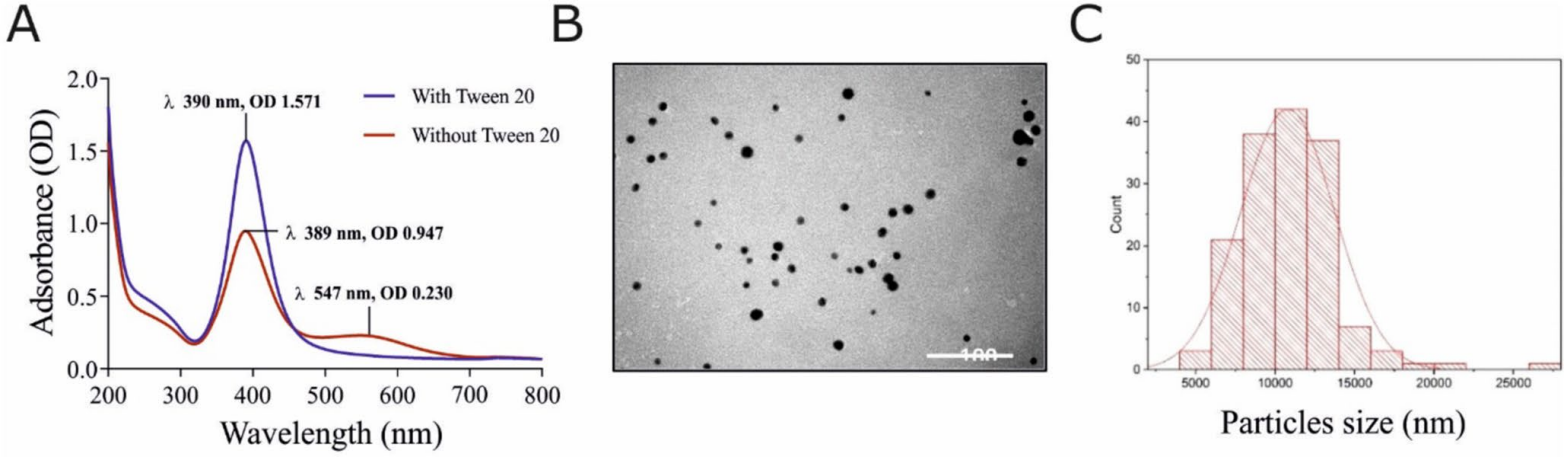
Characterization of AgNPs. (**A**) Representative UV-Vis spectra of newly synthesized AgNPs after centrifugation with 0.025% Tween 20 (blue) and without the stabilizer (red). (**B**) TEM image of AgNPs at 100 000× magnification. Scale bar: 100 nm. (**C**) Average particle size distribution (in nm) of AgNPs based on TEM image analysis

The synthesis of AgNPs was carried out by a modification of the Agnihotri method using water as the medium and citrate as a stabilizing agent [34]. After centrifugation with Tween 20, the nanoparticles were characterized by their plasmonic resonance spectrum and TEM imaging. As shown in Fig. 1A, the suspension exhibits a maximum peak at 390 nm, corresponding to Localized Surface Plasmon Resonance (LSPR). In the absence of Tween 20, AgNPs showed a redshift in their absorbance spectrum, accompanied by a colour change from golden yellow to blue/gray (Fig. 1A), consistent with aggregation. The particle size distribution obtained from TEM analysis (Fig. 1B-C) revealed that the average diameter of AgNPs was approximately 10 nm. This value is consistent with the size derived from the LSPR properties of AgNPs [37].The mean zeta potential was measured to be − 25.1 ± 2.0 mV, corresponding to an electrophoretic mobility of − 1.956 μm·cm/V·s (conductivity: 0.064 mS/cm), indicating moderate electrostatic stabilization of the colloidal suspension (Supplementary Figure S1). Based on the optical properties, the concentration of AgNPs was estimated at 168 ± 27 nM (*n* = 4) [37].

### AgNPs activate a cationic current TRP-like in human glioblastoma U251 cells

**Fig. 2:**
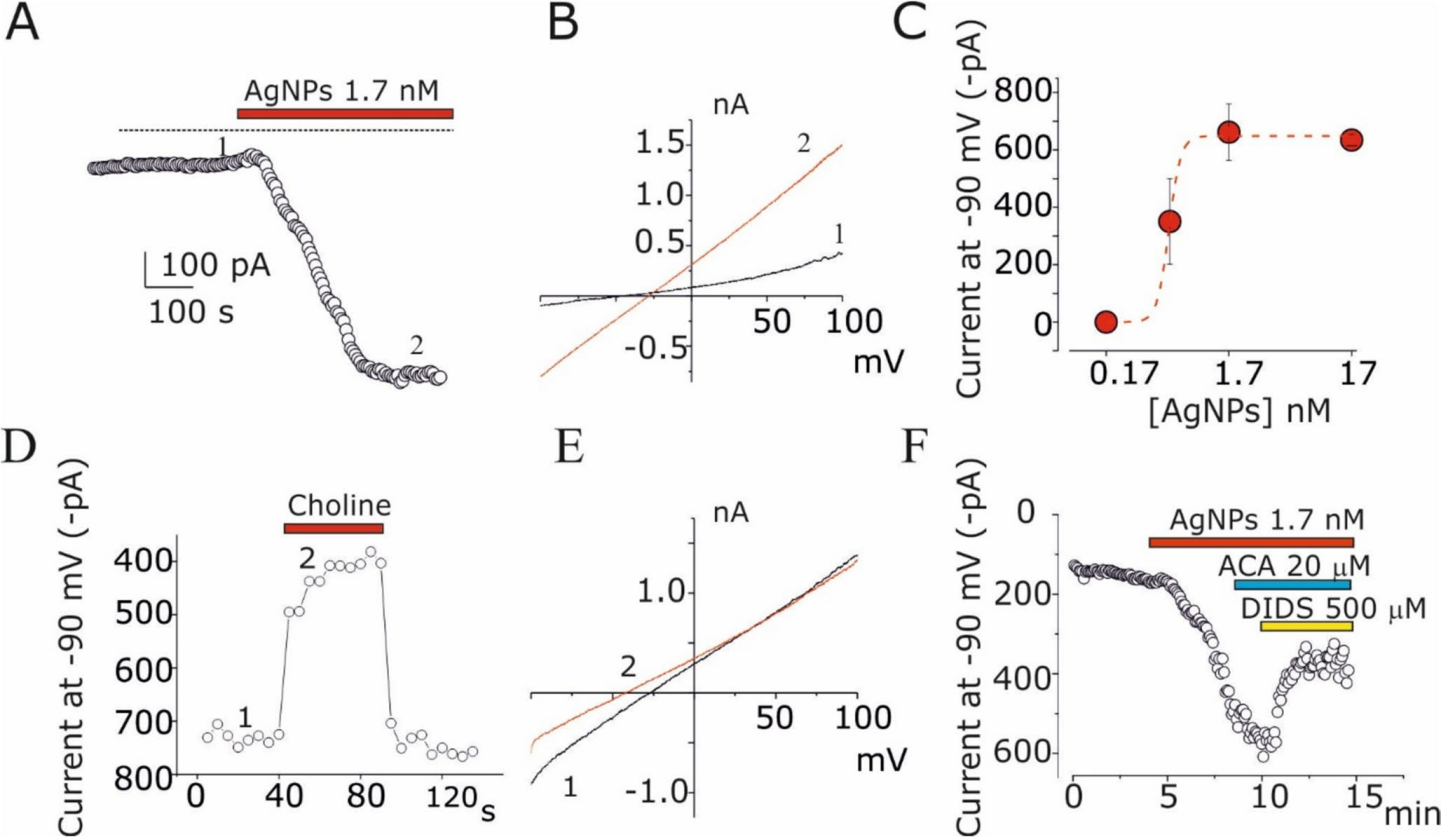
AgNPs activate TRP-like cation currents in U251 cell lines. (**A**) Time course of inward current at −90 mV before and after the application of 1.7 nM AgNPs. (**B**) I-V relationship before and after current activation by AgNPs, corresponding to the time points indicated in A. (**C**) Dose-response relationship of AgNPs-activated currents at various AgNPs concentrations. Dashed lines represent the best fit of the data points using Hill’s equation: 1/1+(IC_50_/[AgNPs])^h^ where IC_50_ is half current activated amplitude and h is the Hill’s number. (**D**) Time course of currents at −90 mV upon replacing external sodium solution with choline. (**E**) I-V relationship obtained by voltage ramps from − 100 to 100 mV before and during sodium/choline exchange in the extracellular bath, at the time points indicated in D. (**F**) Time course of the inward current recorded at − 90 mV before and after the application of 1.7 nM AgNPs, and during the application of 20 µM ACA and 500 µM DIDS, as indicated by the time bars

Patch-clamp technique in whole-cell perforated configuration was used to investigate the electrophysiological effects of AgNPs perfusion in U251 GB cells. A representative time course is shown in Fig. 2A, where each point corresponds to the current at −90 mV recorded during a ramp voltage protocol (−100 mV to 100 mV, Vh = 0 mV), repeated every 5 s during application of 1.7 nM AgNPs. A rapid increase in inward current at −90 mV was observed, reaching a stable activation after a few minutes (Fig. 2A). The mean current recorded in five experiments under identical conditions was 715 ± 142 pA. The I-V relationships before and after AgNPs application (Fig. 2A, B) exhibited a reversal potential of −22.9 ± 4.6 mV (*n* = 5), excluding sodium- or potassium-selective currents (the Nernst potentials for potassium and sodium under these conditions were ~−90 mV and + 65 mV, respectively). AgNPs activated a cationic current in a dose-dependent manner, with an EC_50_ of approximately 0.5 nM (Fig. 2C). Replacing extracellular sodium with choline shifted the reversal potential by ~ 20 mV in a negative direction (Fig. 2D, E), while substituting chloride with gluconate had no effect (data not shown). These findings indicate that the current induced by AgNPs is cationic, nonselective, and permeable to sodium ions, similar to TRPM2-like currents previously reported activated by AgNPs in GB [38]. However, the application of the TRPM2 current blocker N-(p-amylcinnamoyl)anthranilic acid (ACA [38]), at a concentration of 20 µM did not modify the currents activated by AgNPs (Fig. 2F). ACA application did not affect the activation process induced by AgNPs and did not display any blocking effect even at 60 µM (Supplementary Figure S2). 500 µM diisothiocyano-2,2’-stilbenedisulfonic acid (DIDS) partially blocked the cationic currents activated by AgNPs (Fig. 2F), similarly to what observed on IAg in U251 cells and in Xenopus oocytes [35, 40].

### AgNPs promote intracellular calcium increase, membrane blebbing, and mitochondrial depolarization

**Fig. 3:**
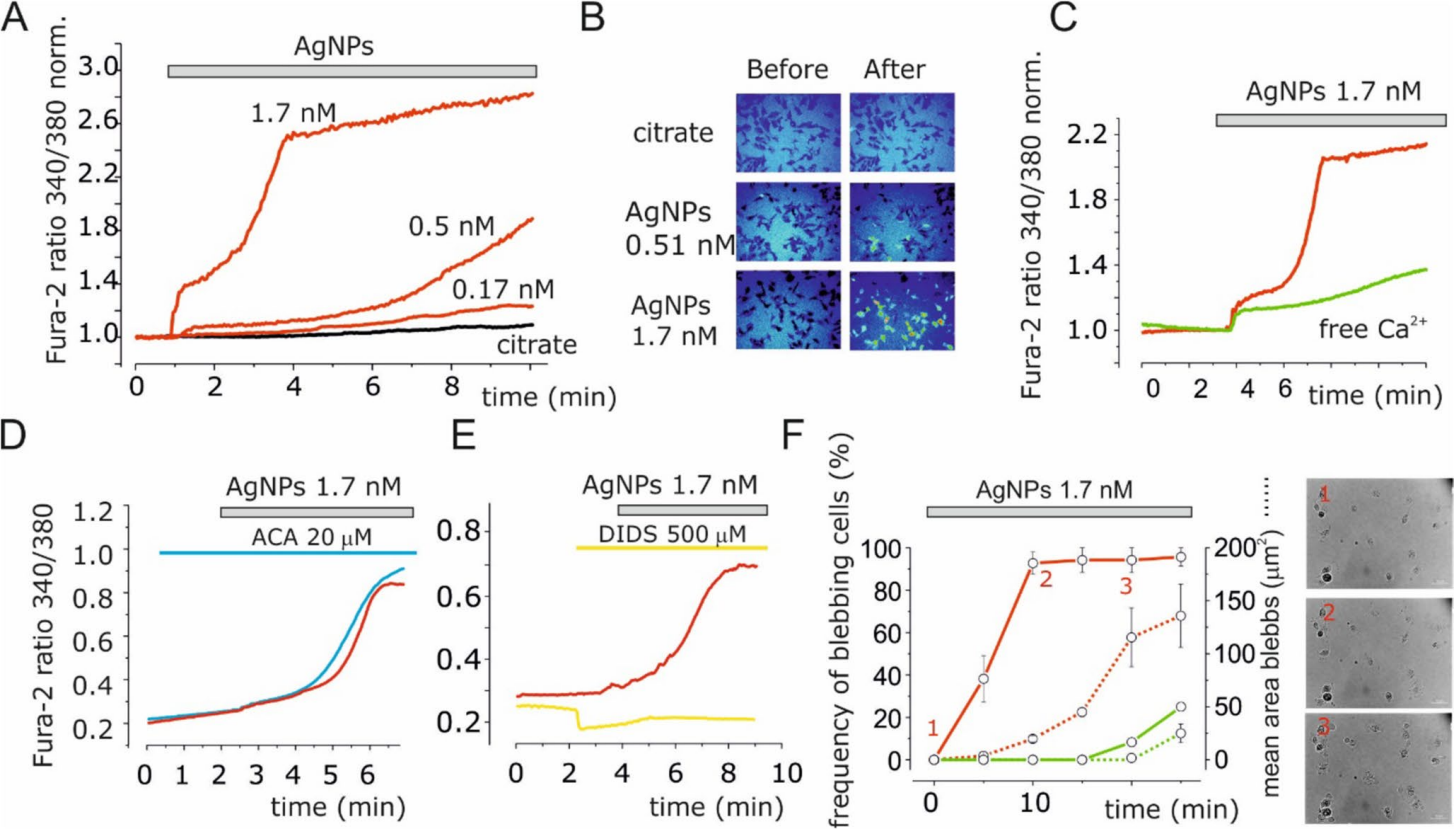
AgNPs promote intracellular Ca^2+^ increase and membrane blebbing in U251 glioblastoma cells. (**A**) Time course of intracellular calcium levels, indicated as fluorescence ratio (340/380 nm excitation) using the ratiometric live-cell dye FURA-2, measured every 3 s in 30 cells before and after AgNPs (or citrate, as control) application at the indicated concentrations. The time of application of AgNPs is indicated by the gray bar. (**B**) Pseudocolor images of the 340/380 fluorescence ratio before (minute 1) and after (minute 8) application of 0.51 and 1.7 nM AgNPs. (**C**) Time course of intracellular Ca^2+^ as FURA-2 emission ratio (340/380) under control conditions (red trace, *n* = 20) and in calcium-free extracellular solution (green trace, *n* = 20). (**D**) Time course of intracellular Ca^2+^ as FURA-2 emission ratio (340/380) under control conditions (red trace, *n* = 39) and in presence of 20 mM ACA (blue trace, *n* = 59). (**E**) Time course of intracellular Ca^2+^ as FURA-2 emission ratio (340/380) under control conditions (red trace, *n* = 61) and in presence of 500 µM DIDS (yellow trace, *n* = 31). (**F**) Membrane bleb formation following 1.7 nM AgNPs treatment in U251 cells. Solid and dashed lines indicate the frequency of blebbing cells (as percentage of total cells) and mean bleb size, respectively. Green lines indicate blebbing behaviour in calcium-free extracellular solution. Inset contains representative images of bleb formation at 0, 10, and 20 min after AgNPs application. Results are presented as mean ± standard error of the mean (SEM)

Since IAg promotes calcium influx [35], the effects of AgNPs on intracellular calcium levels in U251 cells using FURA-2 were investigated. AgNPs application increased intracellular calcium concentration ([Ca^2+^]_i_) in a dose-dependent manner (Fig. 3A, B). A significant increase in [Ca^2+^]_i_ was observed approximately 30 s after application of 1.7 nM AgNPs, with a consistent response observed across all cells. Lower concentrations (0.51 nM and 0.17 nM) induced only minor increases in [Ca^2+^]_i_ (Fig. 3A, B). Bath application of 1 mM citrate had no effect on [Ca^2+^]_i_ (Fig. 3A, B). Removing extracellular calcium prevented the AgNPs-induced increase in [Ca^2+^]_i_ (Fig. 3C), confirming that this rise depends on calcium influx from the extracellular compartment. The AgNPs-induced rise in intracellular Ca^2+^ was not modified by treatment with 20 µM ACA (Fig. 3D), whereas it was inhibited by treatment with 500 µM DIDS (Fig. 3E). At 1.7 nM, AgNPs also induced membrane blebbing (Fig. 3F). Blebs appeared after about 10 min of application, and their size increased over time, reaching an average area of ~ 200 μm^2^ within 20 min (Fig. 3F, red traces). Membrane blebbing formation and growth was inhibited in calcium-free external solution (Fig. 3F, green traces). AgNPs (1.7 nM) caused a rapid decrease in TMRM fluorescence intensity compared to control conditions, consistent with mitochondrial depolarization (Figure S3, black vs. red dots). This depolarization was absent in calcium-free conditions, demonstrating the critical role of IAg activation and calcium influx (Figure S3, green dots).

### AgNPs release silver ions via oxidative reactions involving cellular H_2_O_2_

**Fig. 4:**
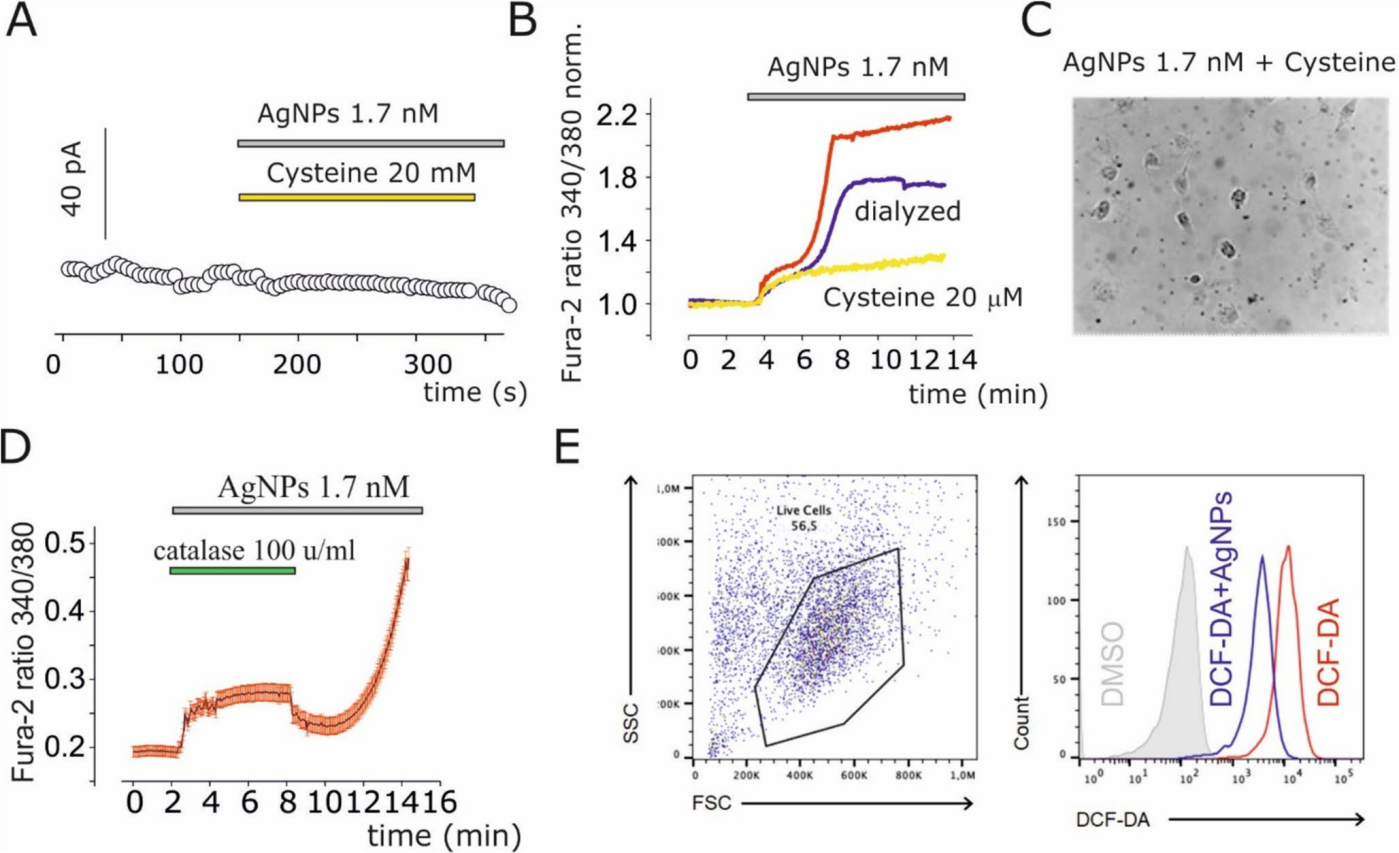
AgNPs effects depend on Ag^+^ release via H_2_​O_2_​ production in U251 cells. (**A**) Time course of currents at −90 mV during AgNPs and cysteine co-application, under conditions described in Fig. 2. (**B**) Cysteine abolished AgNPs-induced intracellular calcium increases, as evaluated by FURA-2 imaging (yellow trace vs. red trace). The blue trace represents calcium elevation induced by dialyzed AgNPs. (**C**) Image taken after 20 min of AgNPs and cysteine co-application, showing no bleb formation. (**D**) Time course of FURA-2 ratio during co-application of AgNPs and catalase, and after catalase removal. Intracellular calcium increase after catalase activity was removed. Note that the apparent increase in the ratio observed during catalase application could be due to a slight emission of the enzyme at the examined wavelengths. (**E**) Flow cytometric analysis of intracellular ROS production following AgNPs exposure using DCF-DA assay. Left, representative FSC (forward scatter) versus SSC (side scatter) dot plot showing the gating strategy to identify the live cell population; right, overlay histograms of DCF fluorescence intensity. Cells treated with vehicle control (DMSO, gray), DCF-DA alone (blue), and DCF-DA + AGNPs (red) are shown

Since AgNPs are known to release Ag^+^, and due to the fact that the activated current recorded in this work share similar properties with IAg [35, 40], the role of thiol-reducing and Ag^+^ chelating agents such as cysteine was investigated. Cysteine (20 µM) completely inhibited AgNPs-induced inward currents (Fig. 4A), intracellular calcium increase (Fig. 4B), and membrane blebbing formation (Fig. 4C). These results suggest that the release of silver ion from AgNPs is responsible for the observed biological effects. To ensure that free Ag⁺ contamination was not involved, we dialyzed the AgNPs solution overnight to remove unreacted silver. Dialyzed AgNPs still induced intracellular calcium increases (Fig. 4B, blue trace). We further hypothesized that Ag^+^ release might result from oxidative reactions driven by cellular H_2_O_2_. Co-application of AgNPs with catalase (CAT) (100 U/mL), an H_2_O_2_-scavenging enzyme, resulted in the abolition of AgNPs-induced calcium increase (Fig. 4D) that was reversible after washout of catalase activity (Fig. 4D). Measurement of intracellular reactive oxygen species, including H_2_O_2_, using DCF-DA dye revealed a significantly reduced intracellular H_2_O_2_ levels in cells following AgNPs administration (Fig. 4E), suggesting that AgNPs may modulate intracellular ROS levels under acute exposure conditions.

### Involvement of KCa3.1 channels in AgNPs-induced radioresistance of U251 cells

**Fig. 5:**
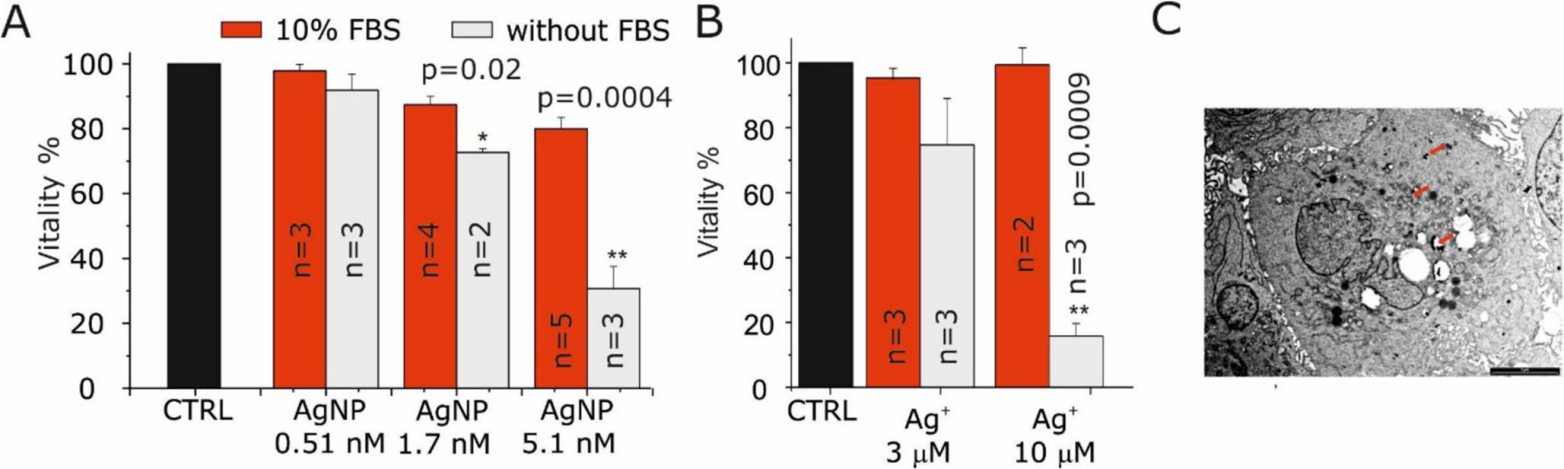
AgNPs and Ag^+^ reduced cell viability in serum dependent manner. (**A**) MTT assay of U251 cells treated with increasing doses of AgNPs for 24 h in normal medium (red bars) and medium without serum and antibiotics (gray bars). (**B**) MTT assay of U251 cells treated with 3 and 10 µM Ag^+^ for 24 h in normal medium (red bars) and medium without serum and antibiotics (gray bars). (**C**) Representative TEM image of U251 cells treated with AgNPs for 24 h, showing AgNPs internalization as dark aggregates in the cytoplasm, mitochondrial membrane, and vesicles (red arrows)

Previous studies demonstrated that AgNPs enhance IR-induced cell death and act as radiosensitizers in GB [14]. To further evaluate their effect on cell proliferation, MTT assays were performed under standard culture conditions. Before, AgNPs stability was assessed in a simulated culture medium (see Materials and Methods) by comparing the ability of freshly dispersed AgNPs and AgNPs pre-incubated for 24–48 h at 37 °C in 5% CO₂ to increase intracellular Ca^2+^ levels. The magnitude of Ca^2+^ responses was not reduced over time, indicating preserved biological activity and supporting the colloidal stability of AgNPs (Supplementary Figure S4). MTT assays showed that AgNPs had no effect on cell viability at 0.51 nM, while a slight but significant decrease in cellular vitality was observed at 1.7 nM and 5.1 nM. (Fig. 5A). As illustrated in Fig. 5A, this effect was more pronounced in the serum-free medium, which is consistent with the findings that the toxicity of AgNPs is modulated by silver serum chelator property [40]. The role of FBS in the medium was further demonstrated when U251 cells were treated with free Ag^+^ ions. In this case, viability remained unaltered due to the presence of the chelating agents. In contrast a pronounced impairment of viability was observed in the absence of the serum (Fig. 5B). The concentration of AgNPs able to evoke significant effects on cell vitality was considerably lower than that required using free Ag^+^ ions. This phenomenon could be ascribed to the efficient internalization of AgNPs in U251 cells, observed 24 h after the treatment (Fig. 5C).

**Fig. 6:**
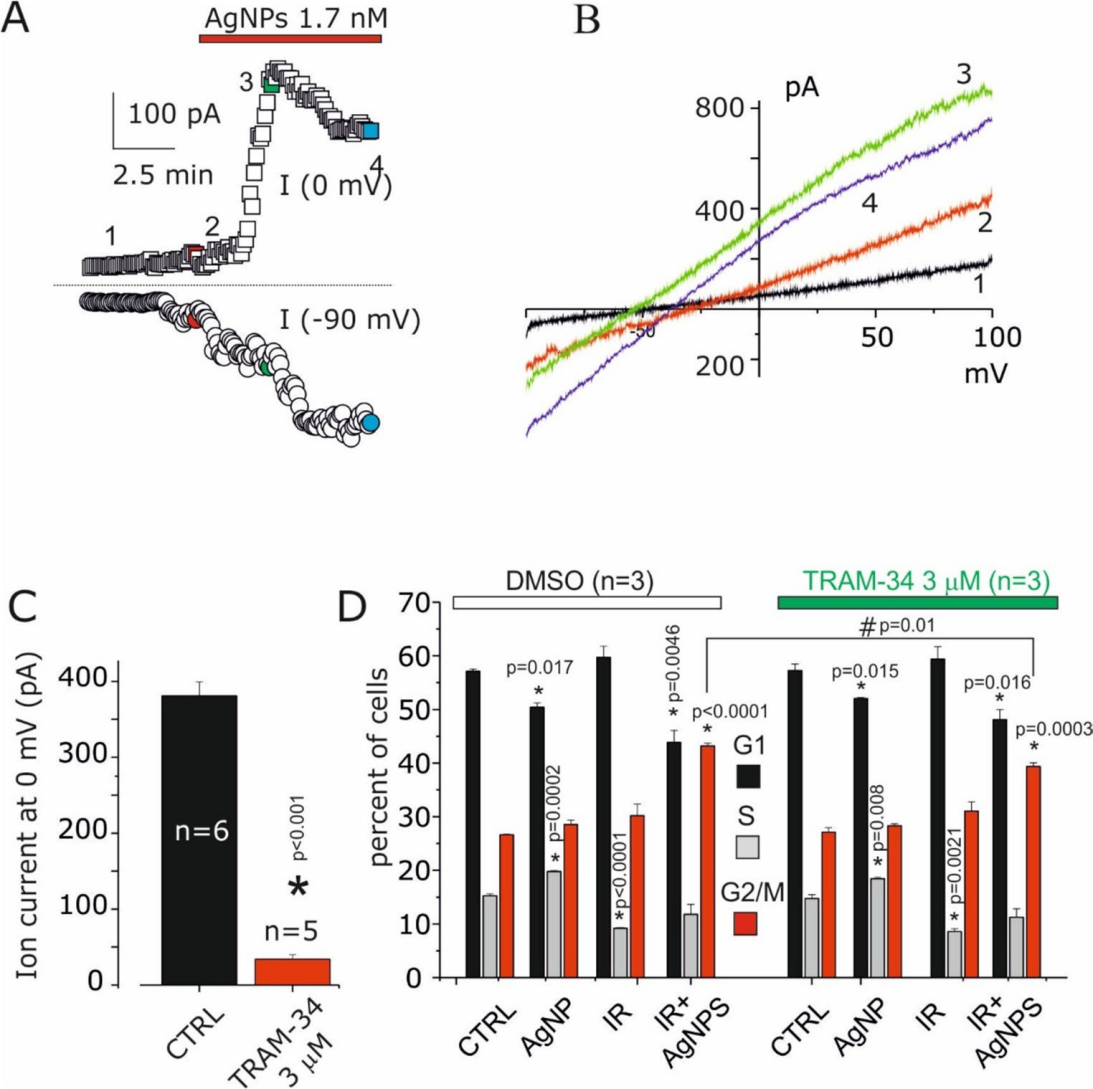
AgNPs activate KCa3.1 currents and participate to G2/M block after radiation co-treatments. (**A**) Time course of inward current at −90 mV and outward current at 0 mV before and after the application of 1.7 nM AgNPs. (**B**) I-V relationship before and after current activation by AgNPs, corresponding to the time points indicated in A). (**C**) Bar plot of currents activated by AgNPs in presence and absence of TRAM-34 (3 µM). (**D**) Cell cycle distribution in control, AgNPs, IR, and AgNPs + IR conditions, with or without the selective KCa3.1 blocker TRAM-34. G2/M accumulation observed during AgNPs + IR co-treatment was reduced in the presence of TRAM-34. * indicates statistical t-test with *p* < 0.05 within the group (DMSO and TRAM-34), whereas # indicates statistical t-test with *p* < 0.05 between groups (DMSO vs. TRAM-34)

Since AgNPs increase intracellular calcium, it was investigated whether this event is capable of activating the intermediate-conductance potassium channel (KCa3.1), which has been implicated in radioresistance processes in vitro and in vivo in GB [28–31]. To this end, we repeated the electrophysiological recordings of AgNPs in the absence of TRAM-34, monitoring membrane potentials at 0 mV and − 90 mV in order to simultaneously detect both the outward potassium current and the IAg current using the same voltage ramp protocol. The dual time course observed following AgNPs application is shown in the representative Fig. 6A. The IAg current (−90 mV) (Fig. 6A and B, red trace) is activated with the same temporal profile reported in Fig. 2A, followed by the activation of an outward current (0 mV) with a reversal potential close to the theoretical equilibrium potential for potassium (Fig. 6A and B, green trace). At later stages, the ensemble of activated currents displays reduced potassium selectivity, consistent with the contribution of the nonselective IAg current (Fig. 6A and B, purple trace). These events at 0 mV were not observed in the presence of 3 µM TRAM-34, where only the IAg current remained detectable (Fig. 6C), indicating that this concentration represents an optimal blocking condition (Supplementary Figure S5). Overall, these electrophysiological effects support the conclusion that AgNPs increase intracellular calcium through Ca^2+^ influx, which subsequently activates the KCa3.1 current. Co-treatment with AgNPs and IR increased G2/M phase arrest, as observed by flow cytofluorimetric analysis (Fig. 6D), likely due to DNA damage [8], according with comet assay (Supplementary Figure S6). TRAM-34, a selective KCa3.1 blocker, reduced G2/M arrest (Fig. 6D), confirming the role of KCa3.1 channels in DNA damage-induced cell cycle arrest during radioresistence [29].

## Discussion

We reported the early effect of AgNPs on electrophysiology, intracellular calcium and morphological imaging in U251 human GB cells. These effects were due to a local release of Ag^+^ on the plasma membrane and activation of IAg that promoted calcium increase, inner mitochondrial depolarization and impaired cell viability. We reported that IAg current is a hallmark of cancer cells, as it is highly expressed in transformed glia compared to normal cells [35]. This current is a non-selective cationic calcium permeable and shows a sensitivity to the chloride current blockers 4,4’-diisothiocyano-2,2’-stilbenedisulfonic acid disodium salt (DIDS), Gd^3+^ and cysteine, as compared to those described in Xenopus oocytes [39]. In DBTRG-05MG GB cell line, AgNPs promoted the activation of TRPM2 channel, based on ACA sensitivity [42]. However, under our experimental conditions we observed an IAg current distinct from classical TRPM2, partially inhibited by DIDS as previously observed [35, 40]. These differences could originate from different AgNPs coating (citrate vs. polyvinylpyrrolidone), treatment (acute vs. chronic), different GB cell lines (U251 vs. DBTRG-05MG). Further experiments are needed to underline the reasons behind these differences. Although not directly addressed in the present study, the possible involvement of additional TRP or chloride channels deserves future investigation to better define the ion channel network engaged by silver nanoparticles and refine the understanding of their mechanism of action. KCa3.1 is expressed in glioblastoma and its high expression is associated with poor prognosis [24, 41]. During irradiation an upregulation of functional expression of KCa3.1 was associated to the increase of migration and invasion [28]. The principal events in KCa3.1 activation induced by IR was the increase of intracellular calcium levels [42]. By using TRAM-34, KCa3.1 current was involved in G1 and G2/M arrest only in IR condition, indicating that the channel is sensible to genotoxic stress [42]. In this context, AgNPs promoted G2/M phase accumulation, reduced cell proliferation and enhanced DNA damage following IR treatment. Disruption of calcium homeostasis plays a major role in pathological and toxicological conditions [42]. Calcium ions have the potential to activate catabolic enzymes like phospholipase, proteases, and endonuclease that further augment the toxicity [43]. In mitochondria Ca^2+^ results in mitochondrial ΔΨ dysregulation, ROS increase, and inhibition of ATP production [42]. The analysis of AgNPs blebbing induction proved that Ca^2+^ can affect also cytoskeleton integrity. Calcium influx mediated by TRP channels modulates cell cycle progression and metabolism in GB cells [44]. Moreover, KCa3.1 was involved in the effects of AgNPs and radioresistance of GB, suggesting a possible positive feedback mechanism which regulates membrane hyperpolarization following intracellular calcium rise [29, 30, 32]. These events are central to radioresistance in GB, since blockade with TRAM-34 reduced the G2/M accumulation induced by AgNPs + IR co-treatment, in agreement with the findings of the Huber group [30]. It has been suggested that KCa3.1 channel blockade may delay the repair of DNA double-strand breaks in G2/M in irradiated glioblastoma cells and, in turn, it could promote the premature re-entry of irradiated cells into mitosis, potentially leading to mitotic catastrophe and reducing the fraction of tumor cells that retains clonogenic capacity [45].

Importantly, the concentration of TRAM-34 used in the present study (3 µM) is supported by our previous pharmacological characterization of KCa3.1 currents in glioblastoma cells. In GL-15 cells, we previously determined a TRAM-34 IC_50_ of 55 nM (Hill coefficient = 0.83) based on dose–response analysis of KCa3.1 current inhibition [25]. Therefore, the concentration of 3 µM used here corresponds to approximately 30-fold the IC_50_, a range commonly employed to ensure near-complete channel blockade in functional studies. The same concentration has been adopted in our subsequent pharmacological investigations of KCa3.1 modulation in human glioblastoma cells [33]. Although off-target effects of TRAM-34 have been reported, including inhibition of cytochrome P450 (CYP) isoforms with IC_50_ values in the micromolar range (≈ 0.9–12.6 µM depending on the isoform) [34], the dose used in the present study remains well supported by prior quantitative pharmacological characterization of KCa3.1 inhibition in glioblastoma models. Nevertheless, additional investigations are warranted when translating these findings to in vivo settings, where systemic administration and complex tumor–microenvironment interactions may amplify potential off-target or immunomodulatory effects. In particular, studies performed in syngeneic glioma models have shown that KCa3.1 targeting with TRAM-34 can influence both tumor cell behavior and microenvironmental components during combined radiotherapy protocols [31], highlighting the importance of careful interpretation when moving toward translational applications [46].

The biological activity of AgNPs depends on many factors including surface area, size, size distribution, shape, coating/capping and agglomeration [47–51]. In this context, we previously reported the application of antibody coated AgNPs as potential therapeutic agents for the treatment of chronic lymphocytic leukemia (CLL) [42]. An additional contribution of our group concerned the characterization of the interaction energies between silver dimers by quantum mechanical calculations, which is the first step in nucleation [52]. Size and, in particular, the surface area/volume ratio is an important factor in this context, as 10 nm citrate-coated AgNPs were found to release 22% as Ag^+^ ions, while 40 nm citrate-coated AgNPs released only 11% as Ag^+^ ions after 24 h in the cell culture media [53]. Here, we demonstrated that serum can reduction of the cytotoxic effect of AgNPs and Ag^+^. We hypothesized that serum may act as Ag^+^ chelators, seizing free silver ions from the medium. The biological effects of silver nanoparticles can be enhanced by strategies for selective targeting, such as conjugation with antibodies, as reported by our group in CLL [42], or integrated with Zr-based metal-organic frameworks (MOFs) UiO-66 for drug delivery [54]. The complete suppression of AgNPs-induced responses by cysteine further supports the notion that bioavailable Ag^+^ mediates these effects, as thiol-containing ligands efficiently sequester silver through the formation of highly stable Ag(I)–thiolate complexes [55], thereby reducing the effective free Ag^+^ concentration below the threshold required to trigger cellular activation. Consistently, in our study, the addition of the thiol-containing chelator cysteine (20 µM) completely abolished AgNPs-induced inward currents, intracellular Ca^2+^ responses, and membrane blebbing, demonstrating that reduction of free bioavailable silver is sufficient to suppress the observed biological effects.

Hydrogen peroxide (H_2_O_2_), although not being a radical species, is a key ROS involved in cellular signaling. In our system, catalase reversibly suppresses AgNPs-induced Ca^2+^ influx, indicating that endogenous H_2_O_2_ is required for AgNPs activity. Within this framework, Ag^+^ generated at the membrane interface may act as the bioactive species responsible for cationic channel activation and the resulting Ca^2+^ influx. The rapid and complete reversibility of the inhibitory effect of catalase after washout (Fig. 4D) suggests that the redox interaction between endogenous H_2_O_2_ and AgNPs occurs predominantly at the extracellular side of the plasma membrane. Our data show that acute AgNPs exposure (within 30 min) leads to a reduction in intracellular ROS levels, as assessed by DCF fluorescence [56]. This decrease can be explained by the local consumption of H_2_O_2_ by AgNPs at the extracellular interface, resulting in its depletion and a consequent increase in its outward transmembrane flux, ultimately lowering the intracellular steady-state levels of H_2_O_2_ and related ROS In addition, AgNPs internalization ‒ observed by TEM imaging at 24 h ‒ may further contribute to intracellular redox modulation, potentially enhancing antioxidant-like effects. Similar behavior has been reported for ultrasmall copper-based nanoparticles in HEK cells, where reduced DCF signals were associated with H_2_O_2_ scavenging activity [57]. Notably, our data reflect early events (within 30 min), where H_2_O_2_ consumption may transiently lower intracellular peroxide levels. At later stages (2–5 h), secondary mitochondrial responses may instead lead to increased ROS production, as reported in previous studies [8]. Further investigations are required to fully elucidate the spatial and kinetic aspects of H_2_O_2_ dynamics during AgNPs effects in our system.

Understanding the interaction between AgNPs and ion channels could lead to the development of therapies modulating their effects to increase the efficacy of radiotherapy as a co-adjuvant in combination with classical drugs such as temozolomide. GB is a currently incurable neoplasm that is resistant to most conventional therapies. Radioresistance, chemoresistance, infiltrative behaviour and the presence of cancer stem cells are among the main characteristics that make this tumour resilient and recurrent. In recent years, many efforts have been made to find alternative ways to treat this tumour, ranging from the improvement of standard protocols, the development of specific targeted drugs or various applications of immunotherapy strategies [58]. Despite these efforts, little progresses have been made and the survival rate of GB patients remains extremely low. In this context, ion channels may represent a new strategy for the treatment of GB thanks to their involvement in many cancer hallmark processes such as proliferation, migration, invasiveness, and radioresistance. However, the translational relevance of these findings must be carefully validated in complex in vivo systems, where tumor–microenvironment interactions, immune components, and systemic pharmacokinetics critically influence therapeutic responses. In particular, syngeneic models represent an essential step to evaluate the impact of ion channel modulation within an intact immune context, which is highly relevant for glioblastoma progression and therapy resistance [29, 31]. The interplay between AgNPs, ion channels, and immune-mediated mechanisms may significantly affect treatment efficacy and safety profiles. Therefore, future studies in orthotopic and syngeneic GB models will be necessary to assess both therapeutic benefit and potential off-target effects. Such investigations will be crucial to bridge the gap between in vitro mechanistic insights and clinically applicable combinatorial strategies.

## Supplementary Information

Below is the link to the electronic supplementary material.

Supplementary Material 1 (DOCX 634 KB)

## Data Availability

All data supporting the findings of this study are included in the manuscript and/or in the Supplementary Information files.
