# Supplementary material for: KCa3.1 mediates radioresistance of silver nanoparticles in human glioblastoma cells
Source: Pflugers Arch. 2026 May 20;478(6):50. doi: 10.1007/s00424-026-03179-8 (PMC13186830; doi:10.1007/s00424-026-03179-8)
Supplement: Supplementary file 1 — Supplementary Material 1 (DOCX 634 KB) [file 424_2026_3179_MOESM1_ESM.docx]

**SUPPLEMENTARY FILE**


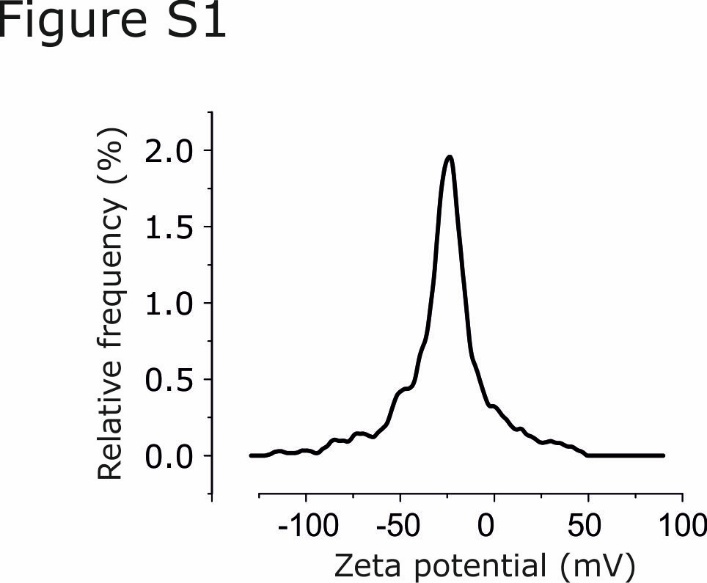


**Figure S1. Zeta-potential of AgNPs.** Representative zeta-potential distribution obtained by electrophoretic light scattering (ELS) at 25 °C (dilution 1:8). AgNPs exhibited a negative surface charge with a mean zeta-potential of −25.1 ± 2.0 mV and a distribution peak at −23.9 mV, corresponding to an electrophoretic mobility of −1.956 µm·cm/V·s (conductivity: 0.064 mS/cm). The observed ζ value indicates moderate electrostatic stabilization of the colloidal suspension.


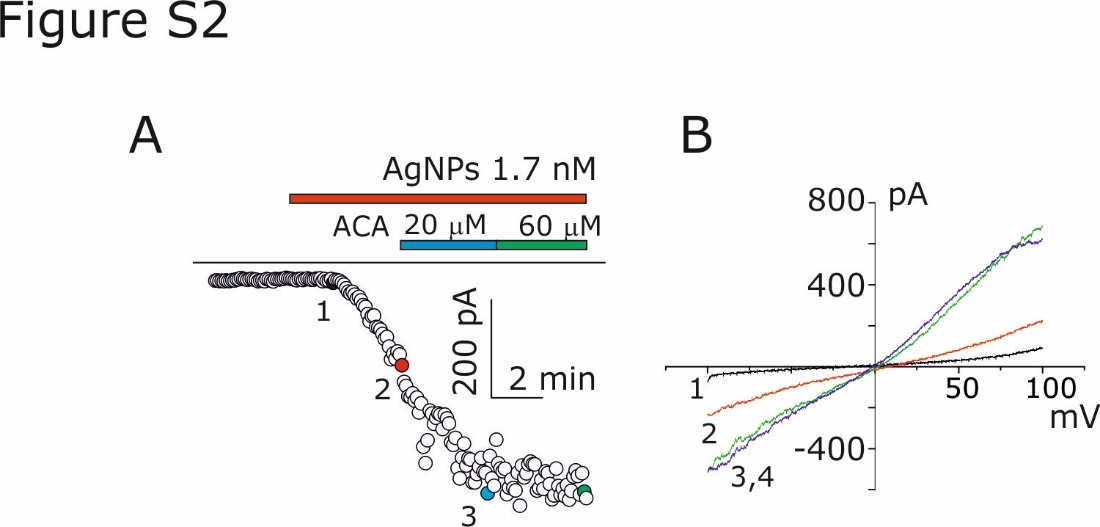


**Figure S2. ACA does not modify IAg current activation by AgNPs. A)** Time course of the inward current recorded at −90 mV before and after the application of 1.7 nM AgNPs, under the pharmacological conditions indicated by the bars at different concentrations of ACA (20 and 60 μM). **B)** I–V relationship before and after current activation by AgNPs under the different pharmacological conditions corresponding to the time points indicated in **(A)**.


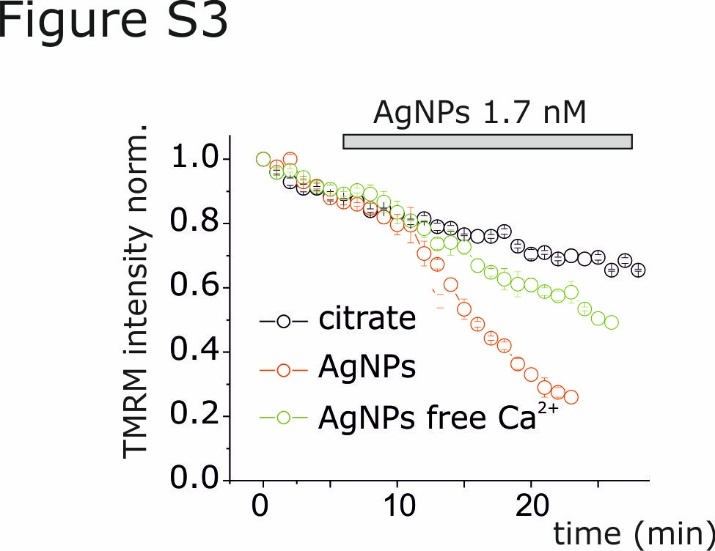


**Figure S3. Effect of AgNPs on mitochondrial polarization.** Time course of mitochondrial depolarization (ΔΨ reduction) following the application of 1.7 nM AgNPs in the presence of extracellular Ca²⁺ (red dots) and under Ca²⁺-free extracellular conditions, showing a reduced effect in Ca²⁺-free conditions (green dots). Black dots represent untreated controls (vehicle, citrate). Results are expressed as normalized means TMRM fluorescence intensity, averaged from at least 50 cells across three independent experiments (n = 3).


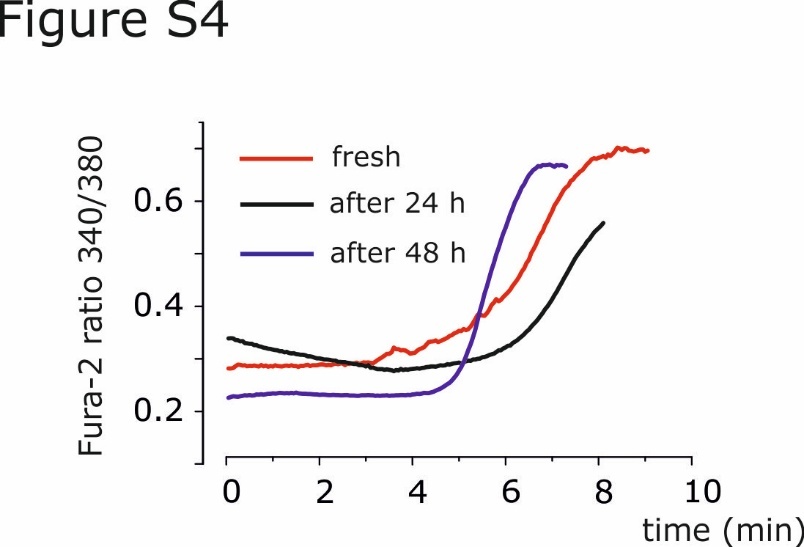


**Figure S4. Colloid’s stability of AgNPs.** Time course of intracellular Ca^2+^ increase in U251 following application of freshly colloid dispersion of AgNPs (red trace, n=58) and after aging at 24h (black trace, n=63) and 48h (blue trace, n=126) in the medium utilised for electrophysiological recordings (see Methods).


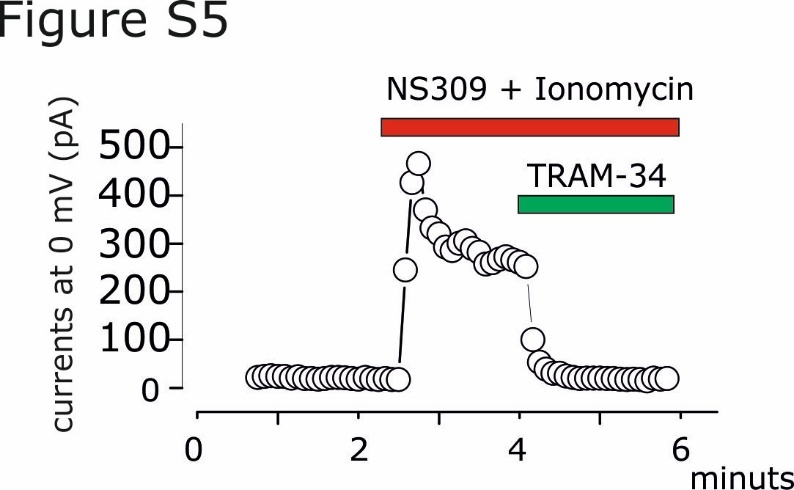


**Figure S5. TRAM-34 (3 µM) completely blocks KCa3.1 current.** Time course of the current measured at 0 mV during voltage ramps from −100 mV to +100 mV applied from a holding potential (Vh) of 0 mV. Ramps were delivered every 5 s in the whole-cell perforated configuration, as described in the experimental conditions (see Figure 2). Experiments were performed in the presence of TEA and octanol to block BKCa channels and gap junctions, respectively. KCa3.1 currents were activated by the application of NS309 (3 µM) plus ionomycin (1 µM), as previously reported (33). Subsequent application of TRAM-34 (3 µM) almost completely abolished the NS309 + ionomycin-activated current.


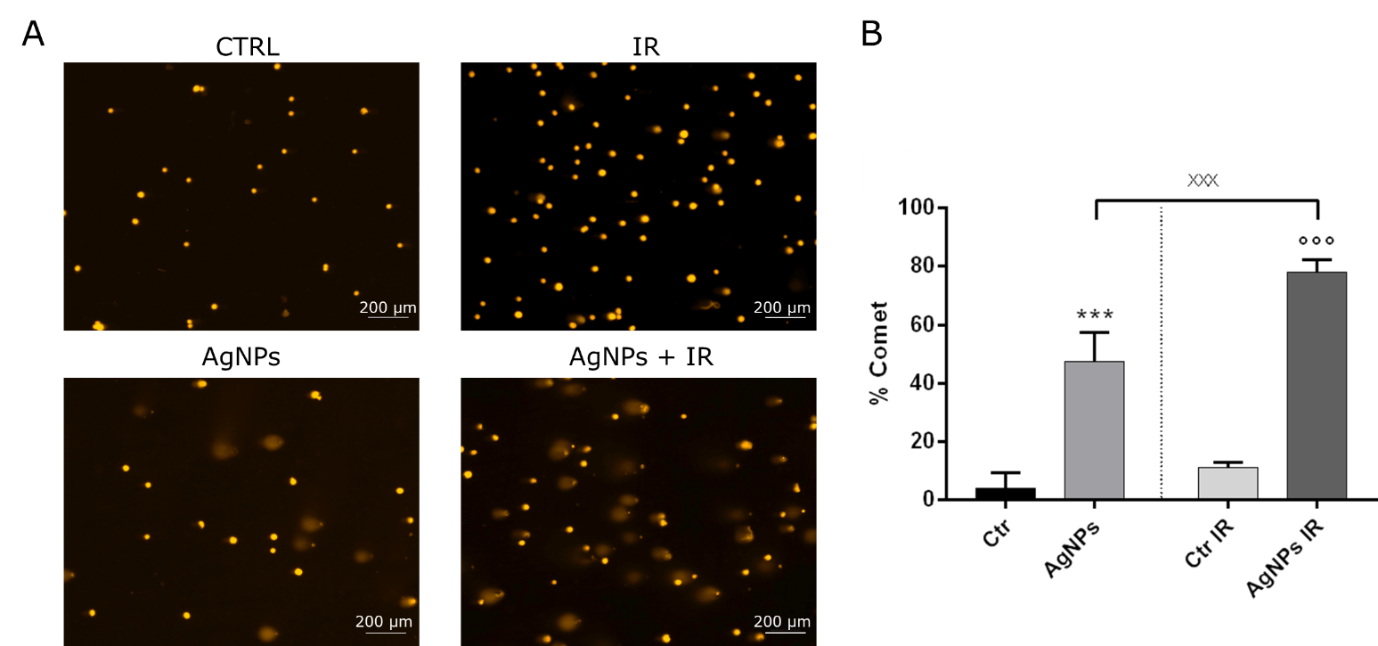


**Figure S6. Enhancement of IR DNA damage by AgNPs.** **A)** Fluorescence images of U251 comet assay after 24 h: control condition (upper left), following 3.5 Gy IR exposure (upper right), after treatment with 1.7 nM AgNPs (lower left), and after combined treatment with 1.7 nM AgNPs and 3.5 Gy IR exposure (lower right). Scale bar represents 200 μm. **B)** Comet percentages of U251 cells in above-mentioned conditions. Columns represent mean percentages of a minimum of 3 experiments. At least 50 cells/experiment were analyzed. Three symbols represent p<0.001, using unpaired t-tes: AgNPs vs control (*), AgNPs irradiated vs control irradiated (°), AgNPs irradiated vs non-irradiated (X).
